# Augmented expression of polo-like kinase 1 indicates poor clinical outcome for breast patients: a systematic review and meta-analysis

**DOI:** 10.18632/oncotarget.17301

**Published:** 2017-04-20

**Authors:** Yunfeng Zhang, Zhibin Wu, Dapeng Liu, Meng Wang, Guodong Xiao, Peili Wang, Xin Sun, Hong Ren, Shou-Ching Tang, Ning Du

**Affiliations:** ^1^ Department of Thoracic Surgery and Oncology, the Second Department of Thoracic Surgery, Cancer Center, the First Affiliated Hospital of Xi’an Jiaotong University, Xi’an, Shaanxi Province, 710061, China; ^2^ The Third Department of Surgery, Shenmu County Hospital, Yulin, Shaanxi Province, 719300, China; ^3^ Georgia Cancer Center Medical College of Georgia, Augusta University, Augusta, GA, 30912, United States; ^4^ Tianjin Medical University Cancer Institute and Hospital, Tianjin 300060, China

**Keywords:** polo-like kinas 1, breast cancer, clinical outcome, meta-analysis

## Abstract

Polo-like kinases 1 (PLK1), a key regulator of mitosis, plays an essential role in maintaining genomic stability. Up-regulation of PLK1 was found in tumorigenesis and tumor progression of diverse cancers. However, the clinicopathological and prognostic implications of PLK1 in breast cancer (BC) have yet to be unveiled. Therefore, using PubMed, Web of Science, Embase, and Chinese databases, we conducted a meta-analysis to define the potential clinical value of PLK1 in BC. Eleven eligible articles with 2481 patients enrolled were included in the present meta-analysis, of which eight studies reported on the relationship between PLK1 expression and clinicopathological features, and nine studies provided survival data in BC patients. Furthermore, the results revealed that high PLK1 levels were significantly associated with larger tumor size (OR=1.703, 95%CIs: 1.315-2.205, *P*<0.001), higher pathological grading (OR=6.028, 95%CIs: 2.639-13.772, *P*<0.001), and lymph node metastasis (OR= 1.524, 95%CIs: 1.192-1.950, *P*=0.001). Moreover, PLK1 was found to be a valuable factor for distinguishing lobular BC from ductal BC with the pooled OR=0.215(95%CIs: 0.083-0.557, *P*=0.002). Analysis of included data showed that high PLK1 expression significantly indicated worse overall survival for BC patients (HR= 3.438, 95%CIs: 2.293-5.154, *P*<0.001), as well as worse cancer specific survival and disease-free survival (HR=2.414, 95%CIs: 1.633-3.567, *P*<0.001 and HR= 2.261, 95%CIs: 1.796-2.951, *P*<0.001, respectively). This quantitative meta-analysis suggests that high PLK1 expression is a credible indicator for the progression of BC and confirms a higher risk of a worse survival rate in patients with BC.

## INTRODUCTION

Breast cancer (BC), the most prevalent cancer overall among women, represents approximately 1.7 million new cancer cases worldwide annually [1]. Due to significant progress made in current treatment, the survival rates of BC patients have increased in developed regions. However, the efficacy of the diagnostic and therapeutic techniques for BC is still limited because of the multi-gene aberrations and complex biological mechanism of this disease. Currently, surgery is still the cornerstone for BC treatment, in conjunction with chemotherapy, radiotherapy, and hormone therapy [2]. Advances in the sequencing of the human genome have facilitated the understanding of the underlying molecular mechanisms of BC heterogeneity [3]. High-throughput molecular profiling has also contributed to a paradigm shift towards increasingly targeted therapy. Detection of proteins and genes, such as estrogen receptors (ERs) [4], vascular endothelial growth receptors (VEGRs) [5] and transcription factor nuclear factor-kappa B (NF-ĸB) [6], involved in BC development at the molecular level could provide insight into the molecular and genetic heterogeneity of BC. Unfortunately, these factors could not clearly unveil the nature of biological changes in BC. Thus, new strategies are needed to explore novel biomarkers for BC to achieve the goal of individualized approaches to treatment.

Polo-like kinase 1(PLK1), the best-characterized member of polo-like kinase family, plays a crucial role in cell-cycle regulation *via* maintaining genome stability [7]. Previous studies showed that PLK1 is involved in the regulation of DNA damage repair by mediating checkpoint kinase 2 (Chk2) and the scaffold protein claspin [8]. Given the crucial role of PLK1 in cell-cycle regulation and DNA damage repair, it is not surprising that it is overexpressed in a variety of malignant neoplasms such as non-small cell lung cancer, bladder cancer, and colorectal cancer [9, 10]. Current studies demonstrated that high expression of PLK1 correlates with poor survival for the patients of gastric cancer and neuroblastomas [11, 12]. However, studies about the clinicopathological and prognostic significance of PLK1 in BC are comparatively few. Thus, we conducted a meta-analysis and systematic review of published literature to investigate the clinicopathological and prognostic implications of PLK1 expression in BC patients.

## RESULTS

### Studies inclusion

**Figure 1:**
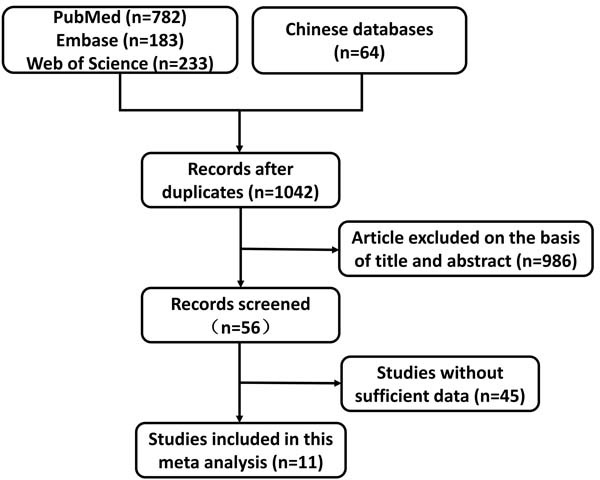
Flowchart of the study selection

Figure 1 shows the flowchart for this meta-analysis. Our search of literature yielded 1042 articles for consideration. After title and abstract evaluation, 56 articles were identified in terms of PLK1 expression in BC patients for further work. Finally, a total of 11 studies enrolling 2481 BC patients ranging from 2005 to 2016 were included [13–23], of which eight studies [13, 15–18, 21–23] reported clinicopathological data and nine studies [13, 14, 16–22] provided prognostic data. The NOS scores of included studies varied from 6 to 9, of which two studies [14, 19] were assesses as 9 scores and two studies [16, 21] were evaluated as 8 scores, as well as 5 studies [13, 17, 18, 20, 22] with 7 scores and one [23] with 6 scores. In total, eight English studies [13, 14, 16, 17, 19–22] and three Chinese studies [15, 18, 23] were included in our analysis, of which the sample sizes ranged from 32 to 979. Specifically, eight studies [13, 15, 16, 18, 21–23] including 1779 patients reported on the relationship between PLK1 and patient age; seven [13, 15–17, 21–23] provided sufficient pathological grading information for 1989 BC patients; seven [13, 15–18, 21–23] provided sufficient lymph node information for 2110 BC patients, and three studies with 1129 patients provided [13, 15, 22] sufficient tumor type information. Regarding crucial biomarkers for BC, studies detecting the association between PLK1 and ER status (seven studies with 1155 patients) [15–18, 21–23], PR status (five studies with 1745 BC patients) [13, 15, 17, 21–23], HER2 status (four studies 1240 BC patients) [13, 15, 17, 22], and p53 mutation status (three studies including 713 patients) [16, 17, 21] were included. Four studies [17–19, 22] exploring the prognostic role of PLK1 for OS in BC patients, as well as three studies [13, 14, 21] for CSS and five studies [14, 16, 17, 19, 20] for DFS. Three studies [14, 18, 19] directly provided the multivariate HR and their 95% CIs; three studies [13, 19, 20] reported univariate HRs; the other five studies did not present estimated HRs; however, we obtained two HRs based on original data in two studies [16, 21] and remaining 3 studies’ HRs [14, 17, 22] were derived from their respective survival curves. See Table 1 for further detailed characteristics of the included studies.

**Table 1:** Characteristics of studies included in the meta-analysis

Author	Year	Region	Patients(n)	Cutoff value	Sample	Assay	Score	Data
Weichert	2005	Germany	135	6(IRS scores)	tissue	IHC	7	Both*
Miller	2005	Sweden	250	6.06	tissue	RT-PCR	8	Both
Ivshina	2006	Singapore	249	6.08	tissue	RT-PCR	8	Both
Han	2007	China	32	6	tissue	IHC	6	Clinicopathological imformation
Loddo	2009	UK	167	0.142	tissue	IHC	9	Prognostic imformation
Li	2009	China	248	1	tissue	IHC	6	Clinicopathological imformation
Li	2011	China	84	NA*	tissue	RT-PCR	7	Both
Ali	2012	UK	979	2	tissue	IHC	7	Both
Maire	2012	France	39	3.94	tissue	RT-PCR	7	Prognostic imformation
King	2012	UK	215	3	tissue	IHC	7	Both
Donizy	2016	Poland	83	8	tissue	IHC	9	Prognostic imformation

NA: not available, Both: study both with clinicopathological and prognostic information.

### Correlation of PLK1 expression and clinicopathological factors of BC patients

**Figure 2:**
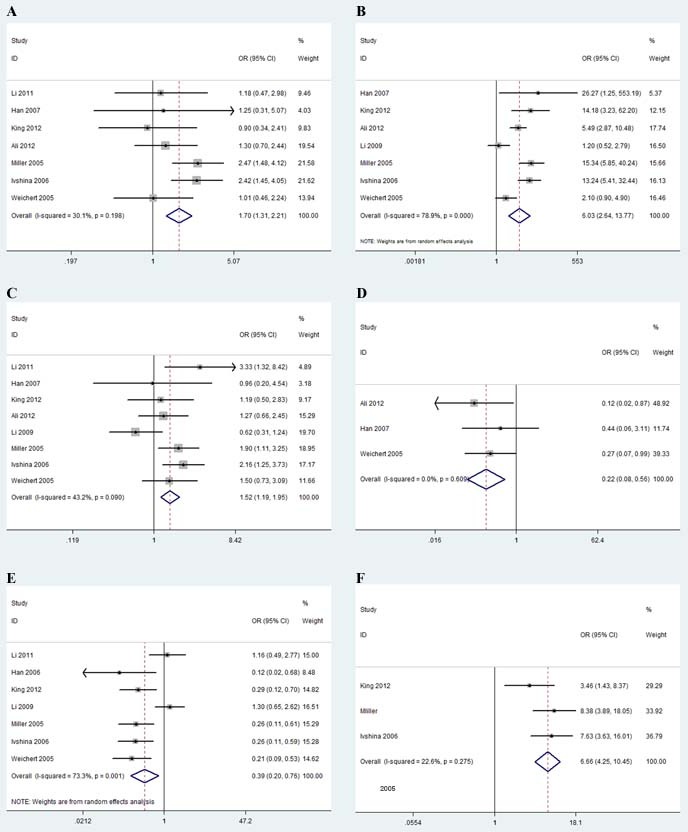
Forest plots of odds ratios for PLK1 expression and clinicopathological parameters in BC patients **A.**Tumor size; **B.** Pathological grading; **C.** Lymph node; **D.**Tumor type; **E.** ER status; **F.** P53 mutation

Results of the meta-analysis indicated that high PLK1 expression significantly correlated with large tumor size (tumor size > 2 cm) with low heterogeneity (OR = 1.703, 95% CIs: 1.315-2.205, *P* < 0.001; I^2^ = 30.10%, *P* = 0.198, Figure 2a) in six studies with 1779 BC patients; higher tumor grade (OR = 6.028, 95% CIs: 2.639-13.772, *P* < 0.001, Figure 2b) in 1989 BC patients; and lymph node metastasis (OR = 1.524, 95% CIs: 1.192-1.950, *P* = 0.001, Figure 2c) in 1975 BC patients.

Also, PLK1 was found to be able to distinguish lobular BC from ductal BC with the pooled OR of 0.215 (95% CIs: 0.083-0.557, *P* = 0.002, Figure 2d). However, there is no significance associated between PLK1 expression and age (OR = 1.018, 95% CIs: 0.795-1.303, *P* = 0.888). Next, we provided evidence that elevated PLK expression was negatively associated with ER-positive status with high heterogeneity in 7 studies (OR = 0.392, 95% CIs: 0.202-0.762, *P* = 0.006; I^2^ = 73.30%, *P* < 0.001, Figure 2e). The pooled OR in 3 studies with 713 BC patients also indicated a strong relationship between elevated PLK1 expression and p53 mutation status (OR = 6.663, 95% CIs: 4.249-10.448, *P* < 0.001, Figure 2f). The pooled ORs did not indicate a significant association between high PLK1 expression with neither PR status nor HER2 status (OR = 0.560, 95% CIs: 0.229-1.364, *P* = 0.202 and OR = 1.447, 95% CIs: 0.795-2.633, *P* = 0.226, Table 2). All the pooled ORs for the association between PLK1 expression and clinicopathological factors are shown in Table 2.

**Table 2:** Main results for meta-analysis between PLK1 and clinicopathological features in breast cancer

Clinicopathological features	Study(*n*)	Pooled OR(95%CIs)	z	*P*	Heterogeneity			Publication bias	
					*I*^2^	*P*	Estimated method	z	*P*
Age	7	1.018(0.795,1.303)	0.14	0.888	0.00%	0.670	Fixed model	0.90	0.368
Tumor size	7	1.703(1.315,2.205)	4.04	<0.001	30.10%	0.198	Fixed model	0.90	0.368
Grading	7	6.028(2.639,13.772)	4.26	<0.001	78.90%	<0.001	Ramdon model	0.90	0.368
Lymph node	7	1.524(1.192,1.950)	3.35	0.001	43.20%	0.090	Fixed model	0.62	0.536
Tumor type	3	0.215(0.083 ,0.557)	3.17	0.002	0.00%	0.609	Fixed model	0.00	1.000
ER status	7	0.392(0.202,0.762)	2.76	0.006	73.30%	0.001	Ramdon model	1.50	1.330
PR status	5	0.560(0.229,1.364)	1.28	0.202	76.90%	0.002	Ramdon model	−0.24	1.000
P53	3	6.663(4.249,10.448)	8.26	<0.001	22.60%	0.275	Fixed model	0.00	1.000
HER2	4	1.447(0.795,2.633)	1.21	0.226	0.00%	0.729	Fixed model	−0.34	1.000

### Prognostic value of PLK1 in BC patients

**Figure 3:**
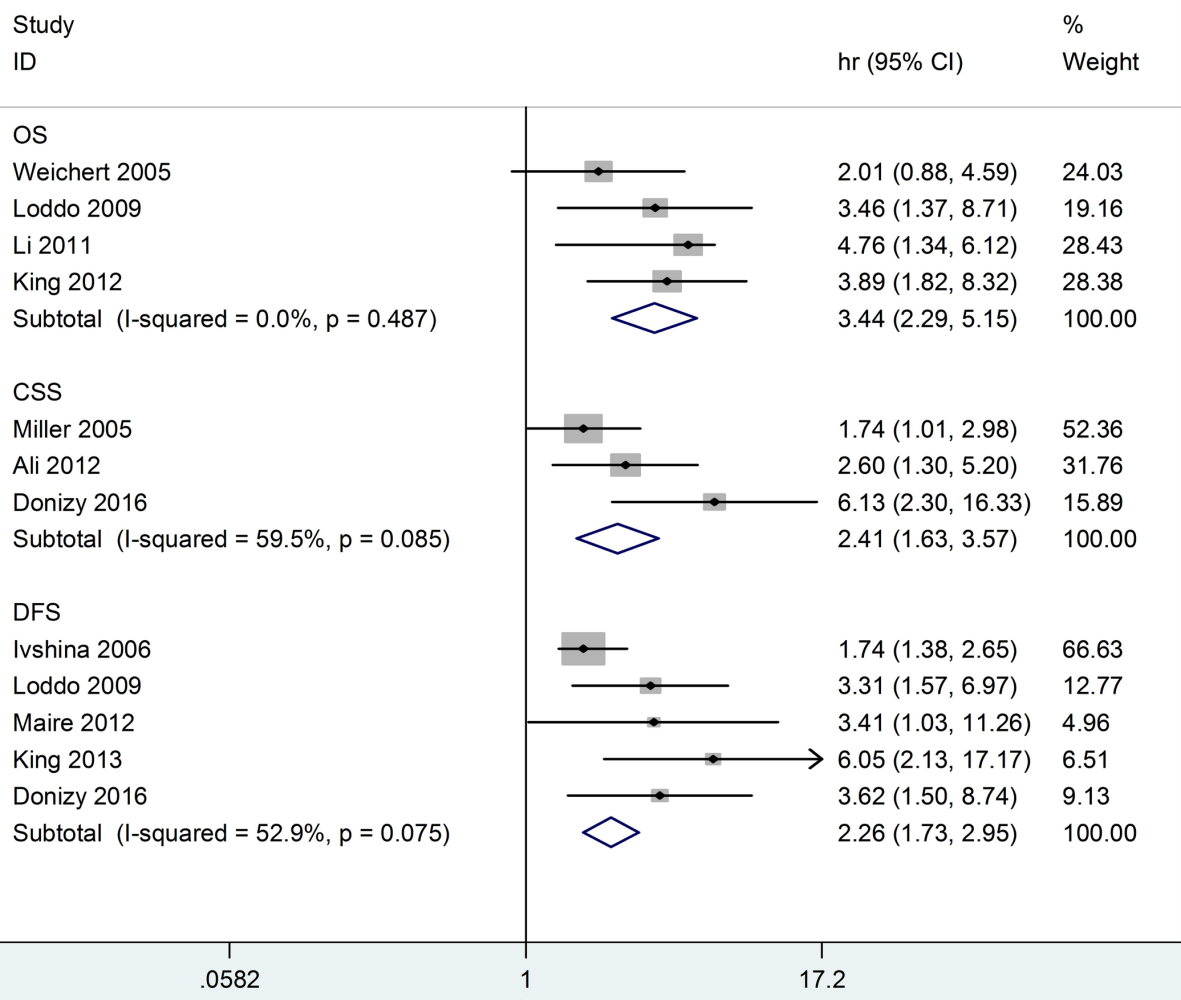
Meta-analysis comparing PLK1 expression and survival in BC patients

To gain insight into the prognostic role of PLK1 in BC, we next investigated the association between PLK1 expression and OS, CSS, and DFS for BC. Consequently, a valuable prognostic effect of PLK1 for poorer OS was found in four studies with a total of 601 BC patients (HR = 3.438, 95% CIs: 2.293-5.154, *P* < 0.001, I^2^ = 0%, *P* = 0.487). The pooled HRs of three studies, including 1312 BC patients, also revealed the predictive effect of high PLK1 expression on shorter CSS (HR = 2.414, 95% CIs: 1.633-3.567, *P* < 0.001; I^2^ = 59.5%, *P* = 0.085,). Meanwhile, high PLK expression also presented an unfavorable factor for DSS in the five studies (HR = 2.261, 95% CIs: 1.796-2.951, *P* < 0.001; I^2^ = 52.9%, *P* = 0.075). We did not conduct a subgroup analysis due to the heterogeneity not being obvious. The combined HRs for the survival analysis are presented in Table 3 and in Figure 3.

**Table 3:** Summary table of HRs and their 95% CI for survival analysis

Survival	HR(95%CIs)	Significance	Method	Publication bias
OS				
Weichert2005	2.010(0.880,4.590)	NS	Survival curve	
Loddo2009	3.460(1.370,8.710)	Poor	Univariate	
Li 2011	4.760(1.341,6.123)	Poor	Multivariate	
King 2012	3.890(1.820,8.320)	Poor	Survival curve	
Combined HR	3.438(2.293,5.154)	z = 5.98, *P* < 0.001; *I*^2^ = 0%, *P* = 0.487	Fixed-effects model	z = 1.02, *P* = 0.308
CSS				
Miller2005	1.739(1.014,2.985)	Poor	Original data	
Ali 2012	2.600(1.300,5.200)	Poor	Univariate	
Donizy 2016	6.130(2.300,16.330)	Poor	Multivariate	
Combined HR	2.414(1.633,3.567)	z = 4.42, *P* < 0.001; *I*^2^ = 59.50%, *P* = 0.085	Fixed-effects model	z = 1.04, *P* = 0.296
DFS				
Ivshina 2006	1.736(1.378,2.646)	Poor	Original data	
Loddo2009	3.310(1.570,6.970)	Poor	Multivariate	
Maire 2012	3.410(1.030,11.260)	Poor	Univariate	
King 2013	6.050(2.130,17.170)	Poor	Survival curve	
Donizy 2016	3.620(1.500,8.740)	Poor	Survival curve	
Combined HR	2.261(1.732,2.951)	z = 6.00, *P* < 0.001; *I*^2^ = 52.9%, *P* = 0.075	Fixed-effects model	z = 0.73, *P* = 0.462

### Test of heterogeneity

In this meta-analysis, we found that there is also obvious heterogeneity in the studies evaluating the association between PLK1 expression and ER status (I^2^ = 73.30%, *P* = 0.001%), as well as PR status (I^2^ = 76.9%, *P* = 0.002). Significant heterogeneity existed in the correlations between high PLK1 expression and tumor grading (I^2^ = 78.90%, *P* < 0.0001). Additionally, studies investigating the prognostic value of PLK1 expression did not present obvious heterogeneity (I^2^ = 0% for OS, *P* = 0.487; I^2^ = 59.5% for CSS, *P* = 0.085; and I^2^ = 52.9% for DFS, *P* = 0.075). The results of the test of heterogeneity for all the analyses are shown in Table 2 and Table 3.

### Publication bias

**Figure 4:**
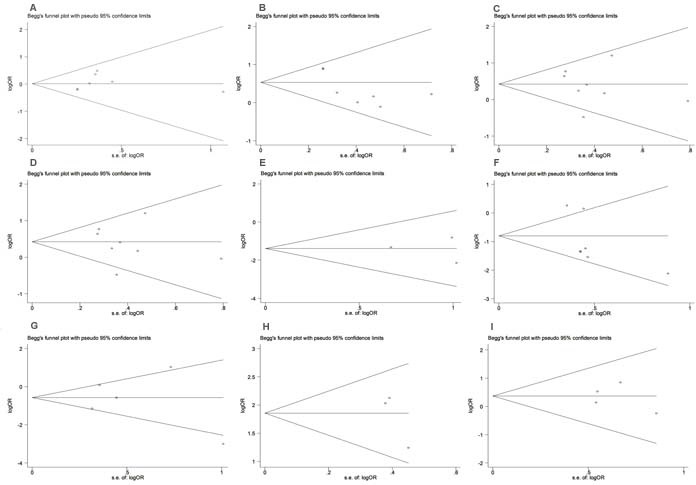
Funnel plot for the publication bias test of the IDH mutations and clinicopathological parameters of BC patients **A.** Age; **B.**Tumor size; **C.**Pathological grading; **D.**Lymph node; **E.**Tumor type; **F.** ER status; **G.** PR status; **H.** P53; **I.** HER2.

As shown in funnel plot, there is no obvious publication bias among all the analysis of PLK1 expression and clinicopathological parameters of BC patients (Figure 4). The results of Begg’ test for each analysis were also presented in Table 2. Nevertheless, the funnel plots and Begg's test did not reveal obvious evidence among all the analysis of PLK1 expression and survival(OS, *P* = 0.308; CSS, *P* = 0.296; DFS, *P* = 0.462;Figure 4 and Table 3).

**Figure 5:**
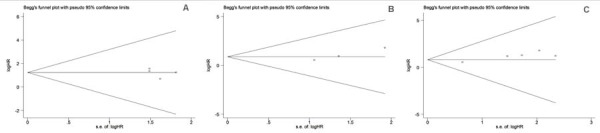
Funnel plots of studies evaluating the PLK1 expression and survival in BC patients A.OS; B.CSS; D.DFS

## DISCUSSION

This meta-analysis investigating the correlation between PLK1 and BC was designed with two objectives. We first compared PLK1 expression with clinicopathological features. PLK1 expression was found to be significantly associated with tumor size, lymph node status, and pathological grading as well as with ER and p53 status, both important factors in BC. Next, we examined the prognostic value of PLK1 expression in terms of BC patient survival. Altogether, our results showed that PLK1 overexpression confers a strongly predictive factor for progression and prognosis in BC.

PLK1, a key regulator of mitotic entry, is considered to be an oncology target in several tumor types, including BC [24–26]. Previous evidence demonstrated that PLK1 overexpression is closely associated with the cell cycle and peak expression of PLK1 was found to occur in the G2/M phase in *in vitro* models [27]. Growing bodies of evidence have revealed that alteration of PLK1 is strongly associated with aneuploidy and mitotic defects, resulting in tumorigenesis by inhibiting Rb and p53 genes [28]. Consistent with our results, previous studies have concluded that PLK1 negatively interacts with the ER and regulates the ER target gene in BC patients. Interestingly, both PLK1-co-activated and ER-targeted genes were enriched in developmental function and act as tumor suppressor factors [29], suggesting a potential interaction of PLK1 with ERs. Moreover, high PLK1 expression was positively associated with p53 mutant status, which is a potent transcription factor in tumor progression. It has been suggested that PLK1 might induce tumorigenesis by downregulating p53 [30]. In a previous study, researchers showed that PLK1 is likely to be a potential target of p53 in DNA damage and directly repressed expression by p53 [31] [35]. Previous evidence also revealed that PLK1 acts as a critical component of the G2/M checkpoint and was inhibited in a p53-dependent manner in tumor cells [32]. Nevertheless, the mechanism explaining how p53 status influences PLK1 expression in BC is uncertain.

Although this meta-analysis enrolled 2481 BC patients overall, several inherent limitations still exist. First, the number of patients in the included studies (especially in OS and DSS studies) is typically small, which decreases the reliability of our results. Second, variability in detection of PLK1 expression and subsequent cut-off value selection introduces a potential source of bias. Lack of a standard threshold in practice, the cut-off value of PLK1 detection varied from 2% to 8%, contributing to the potential heterogeneity. Though the semi-quantitative scale of the score(IRS) was widely used to detect the PLK1 immunohistochemical reaction [33], the cut-off value for high PLK1 immunoreactivity were not concordant. The high PLK1 immunoreactivity were less concordant in the included studies. King et al evaluated PLK1 overexpression by the cutoff of 3, whereas Ali et al used a cutoff value of 1 to define high PLK1 level of BC [13, 17]. Third, several studies detected PLK1 by immunohistochemistry, while other studies used qRT-PCR. Especially, three of the included studies evaluated the PLK1 expression by using microarray profile [16, 20, 21], whereas several studies performed the immunohistochemistry to observe the PLK1 level, leading to the methodological differences. Another key limitation in our study is the method for estimating the HR from KM curves by Engauge, developing an unavoidable decrease of reliability. Fourth, only eligible English and Chinese studies were included; therefore, some qualified studies of other languages were excluded, increasing the potential biases for this meta-analysis. Additionally, several studies did not report a multivariate HR but only a univariate HR. They could not provide an independent, accurate prediction of PLK1 expression because of the multiple molecular abnormalities associated with BC.

In summary, this meta-analysis supported the conclusion that PLK1 expression confers a useful predictive factor for larger tumor size and positive lymph node status, in addition to higher tumor grading. More importantly, high PLK1 expression was closely associated with ER-positive BC and with BC having mutant P53. Of note, we also confirmed that high PLK1 expression markedly shortened OS as well as DFS and DSS for BC patients. However, further well-designed studies enrolled with large cohort patients are needed to define the authentic clinicopathological and prognostic value of PLK1 for BC patients.

## METHODS AND MATERIALS

### Search strategy and selection criteria

An electronic literature search was carried out in the PubMed, Web of Science, and Embase databases (up to 31 October 2017) by using the following MeSHs: “(polo-like kinase 1 OR PLK1 OR serine/threonine-protein kinase 13 OR STPK13) and breast cancer.” Meanwhile, we also searched the eligible studies in Chinese databases, including China Biology Medicine disc (CBM), Chongqing VIP, China National Knowledge Infrastructure (CNKI), and Wan Fang Data. Additionally, we also searched the references of all the studies and bibliographies of other pertinent articles to find related articles.

The meta-analysis considered the following as inclusion criteria: (1) proven diagnosis of BC in humans; (2) evaluation of the relation between PLK1 expression and clinicopathological features or prognosis of BC patients (overall survival (OS), cancer specific survival (CSS), and disease free survival(DFS)); (3) sufficient data to evaluate the odds ratio (OR) or hazard ratio (HR) and their 95% confidence intervals(CIs); and (4) written in Chinese or English. Exclusion criteria for this meta-analysis were the following: (1) reviews, conference abstracts, case reports, or letters; (2) insufficient data to estimate HRs or ORs and 95% CIs; and (3) overlapping articles.

### Data extraction and quality assessments

For included studies, two investigators (Yunfeng Zhang and Zhibin Wu) extracted data as follows: author name, publication year, region, sample size, cut-off value, method of PLK1 detection, calculation methods for HRs, clinical features, and survival data. Thereafter, two researchers independently assessed the quality of each study according to the Newcastle-Ottawa Quality Assessment Scale (NOS) [34]. The study considered high-quality must reach 6 or higher NOS score and the study with less 6 NOS score was considered as low-quality. Any controversies were arbitrated by the third researcher (Dapeng Liu).

### Statistical analysis

ORs and their 95% CIs were pooled to evaluate the association between PLK1 level and clinicopathological features including age (old *versus* young), tumor size (≥2 cm *versus* < 2 cm), pathological grading (G3 *versus* G1-2), tumor type (lobular BC *versus* ductal BC) and lymph node status (yes *versus* no). The correlations of PLK1-positive and ER status (positive *versus* negative), PR status (positive *versus* negative), and p53 and HER2 mutation status (mutation *versus* wild-type) were also estimated by the pooled ORs and their 95% CIs. For survival analysis, we directly extracted HRs and their 95% CIs from the articles when data was available; otherwise, we estimated the HR based on the original data by univariate cox analysis or from the Kaplan-Meier (KM) curves according to the method described by Parmar [35]. Once both multivariate and univariate HRs and 95% CIs were provided for the same cohort of patients, we preferentially obtained HRs from the multivariate analysis due to the influence of multiple factors on the survival outcome. The HR was considered as statistically significant if it did not overlap 1. An observed HR > 1 suggested that high PLK1 expression implied worse survival for BC patients. The Chi-square-based Q statistical test was conducted to evaluate the heterogeneity across the studies. Higgins I^2^ was employed to estimate the degree of heterogeneity and I^2^ larger than 50.0% was considered as obvious heterogeneity. The random-effects (DerSimonian and Laird method) were used when the P value was less than 0.05; otherwise, the fixed-effects model (the Mantel-Haenszel method) was performed [36]. At the end, we evaluated publication bias by using Begg's rank correlation and the funnel plot [37]. A statistically significant two-way P value must be less than 0.05. All statistical analyses were performed by STATA version 12.0 software (Stata Corporation, College Station, TX).
